# Pyelitis in Infancy; a Report of a Case and Review of Current Literature

**Published:** 1916-09

**Authors:** John F. Jenkins

**Affiliations:** Opelika, Ala.


### Journal-Record of Medicine

Successor to Atlanta Medical and Surgical Journal, Established 1855
and Southern Medical Record, Established 1870

**OWNED BY THE ATLANTA MEDICAL JOURNAL COMPANY**

PUBLISHED MONTHLY

*Official Organ Fulton County Medical Society, State Examing Board, Atlanta, Birmingham and Atlantic Railroad Surgeon's Association, Chattahoochee Valley Medical and Surgical Association, Etc.*

A. W. STIRLING, M. D., C. M., D. P. H.; J. S. HURT, B. Ph., M.D.
GEO. M. NILES, M. D., W. J. LOVE, M. D., (Ala.); Associate Editors
E. W. ALLEN, Business Manager

**COLLABORATORS**

W. F. WESTMORELAND, M. D., General Surgery
F. W. McRAE, M. D., Abdominal Surgery
ALLEN H. BUNCE, Medical Societies
E. B. BLOCK, M. D., Diseases of the Nervous System
MICHAEL HOKE, M. D., Orthopedic Surgery
CYRUS W. STRICKLER, M. D., Legal Medicine and Medical Legislation
E. C. DAVIS, A. B., M. D., Obstetrics
E. G. JONES, A. B., M. D., Gynecology
ALLEN H. BUNCE, M. D., Pathology and Bacteriology
R. T. DORSEY, Jr., B. S., M. D., Medicine
L. M. GAINES, A. B., M. D., Internal Medicine
GEO. C. MIZELL, M. D., Diseases of the Stomach and Intestines
L. B. CLARKE, M. D., Pediatrics
EDGAR PAULLIN, M. D., Opsonic Medicine
THEODORE TOEPEL, M. D., Mechano Therapy
R. R. DALY, M. D., Diseases of the Eye, Ear, Nose and Throat
E. G. BALLENGER, M. D., Diseases of the Genito-Urinary Organs

VOL. LXIII. ATLANTA, GA., SEPTEMBER, 1916 NO. 6

### PYELITIS IN INFANCY; A REPORT OF A CASE AND REVIEW OF CURRENT LITERATURE.

By John F. Jenkins, M. D., Opelika, Ala.

Some two years ago I was called in consultation to see an eight months' breast-fed baby [girl], in whom the medical attendant had made the clinical diagnosis of malaria, but the case had not responded to the quinin treatment. The history was about as follows—a previously healthy baby was taken sick with a chill and a remittent type of fever for three or four days, then an afebrile for two or three days, when the fever would recur despite the rather heroic use of quinin. The child was in the third recurrence when seen by myself, and seemed considerably prostrated but still well nourished, in fact, she had taken the breast fairly well all along and the bowel function had been good except for constipation, which had been met by the use of castoria, the pulse and respiration were in accord with the rise and fall of the temperature, the chest was negative on examination, the abdomen was soft and pliable with no complaint of tenderness, the ear drums looked normal. The baby just had a recurring fever and would lay and sleep if not disturbed. Inasmuch as it was in a neighborhood where malaria was not infrequent, the attendant doctor, after excluding the lungs, the throat, the bowels, the ears, had come to the diagnosis of malaria inasmuch as it had begun with a chill and was followed by a remittent fever, which at first subsided under quinin, but again recurred and again recurred under quinin.

I felt that the doctor had clinically made a good diagnosis and had heroically applied the remedy, so it was up to me to do something else. A. L. C. and a differential was made. The L. C. was 24000. The differential was 90 per cent. Pollyneuclear and malarial plasmodium was of course not found. That showed us that we had an infection somewhere, but in the chest, the throat, the ears, the abdomen there was nothing to account for it. The mother said it would be difficult to get a urinary specimen, but we had the child's parts well bathed then set over a clean cup and made pressure over the bladder and got some near a half cup of urine. It was acid in reaction, faintly positive for albumen, and miscroscopically was loaded with non-acid fast bacilli, some pus and epithelial cells.

Well, our diagnosis was then easy, a colon bacillus infection of the kidney pelvis. The child was given water by mouth and by rectum, hexamethylaanamine in gr. ii doses every four hours and fever subsided and did not recur. The treatment was kept up for some weeks intermittently.

This case called my attentions strikingly to the danger of overlooking these kidney infections and the necessity of microscopical examination of the urine in the unexplained fevers of childhood. Therefore, in considering a topic for a paper, it occurred to me that a report of this case and then a review of current literature on Pyelitis in Infancy would be of interest to you, more than any finding of my own.

I will take up the review as follows:

1st. Mode of infection,
2nd. Clinical symptoms,
3rd. Urinary findings,
4th. Duration of Disease,
5th. Treatment.

Mode of Infection. Thomson in an excellent paper based on seventy-five cases of colon infection of the urinary tract in his private practice says:

"It is scarcely ever possible to decide in any individual case which route the bacilli takes in passing from their original harmless position to where they ultimately set up disease. It is, however, quite certain that they are sometimes carried there by the blood stream, sometimes passed by the lymphatic channels, and sometimes ascend from outside by the lumen of the urinary tract.

When the bacilli reach the urinary tract, they are either washed out immediately, or else destroyed; or they may remain for a time doing little or no harm. If the virulence of the organisms has been increased by morbid processes in the intestines, or otherwise, or the normal resistance of the tissues lowered by local or general disease; or perhaps if there has been some retardation of the flow of urine, then more or less violent inflammation may be set up."

He then analyzes the sex incedence of the disease, making the following points:

"1. The number of girls is much greater than that of the boys; among my cases the girls formed 79 per cent.

2. In the young infant the disease begins more often with diarrhoea than in older children. Diarrhoea is more often a marked symptom in boys than in girls. That is, cases setting in during apparent good health are commoner in girls than in boys.

3. Further, during the first six months of life far more boys than girls are affected; although after the sixth month girls largely predominated. When we analyze the ages and sexes of 224 babies under 2 years taken at random from thirteen authors, we find that there is still a considerably greater proportion of boys during the first six months than at any later age.

The extreme rarity of rigors at the onset in boys it noteworthy when compared with the frequency of this symptom in girls.

5. Mild cases of bacillus coli infections are rare in boys in my experience. In male patients the attacks of pyelitis are apt to be severe, and there is usually in them a much larger proportion of cases of fatal pyelonephritis than among girls."

In discussing these five points he writes:

"The great preponderance of female patients seems to force us to the conclusion that ascending infection by way of the uretha must be a common occurrence, and the excess of girls over boys probably represents the frequency with which urethral infection takes place. It seems very unlikely that the colon bacillus can force its way up the lumen of the male uretha. The greater prevalence of antecedent diarrhoea in boys would probably cease to exist if we were able to subtract from the list of girls the primary cases infected per uretham.

The frequency of pyelonephritis in male (the) sex may be explained by the infection in them having usually passed straight from the bowels to the kidney and pelvis, and not having ascended from below. The fact that rigors are so very rare in an acute pyelitis in boys and in pyelonephritis in both sexes, while they are so common in acute pyelitis in girls (in whom we suspect ascending infection) suggest the idea that the ureters may be the particular portion of the urinary tract from irritation of which a rigor most readily arises. The frequency with which rigors are met with in urethral calculus may be held to support this view."

Somewhat similar views are held by Wyman, who from a study of 65 cases at the Children's Hospital in Boston, concludes that ascending route is a common one in girls, and the hematogenous in boys. An analysis of the age and sex of his patients gives the following:

Males under 2 years..............=37%
Females under 2 years ...........+63%
Males over 2 years ................Lo%
Females over 2 years ............=90%

The views of F. H. Smith are less clear; he writes that "in the younger children and babies the infection is apt to be the colon bacillus, and most often from direct fecal contamination of the vulva, or possibly, by more direct route through blood or lymph paths to the kidney, upset bowels, preceding the illness being highly suggestive of increased toxicity of the intestinal flora." In older children, however, he considers that "repeated attacks of acute tonsillitis, acute bronchitis and 'kidney trouble' in sequence, suggest their interdependence."

Freeman believes in the ascending road of infection, because of the preponderance of female cases.

Green, however, writes that pyelitis "probably proceeds by lympatic extension from the intestines," while "acute inflammatory nephritis is most usually hemotogenous in origin. Nevertheless he concludes as predisposing causes "calculi, constipation, phimosis, anal fissues and foci of infection elsewhere."

Cannata and caronia emphasize the necessity of preventing the early infections. Habitual constipation is dangerous because there is usually elimination of bacteria by way of the urinary apparatus. Fecal contamination of the vulva, phimosis and foreign bodies in the bladder, are sources of ascending infections. Chilling, or any trauma of the bladder, is likely to prove the exciting cause. Stiner also mentions the danger of chilling. Langstein has reported two cases of pyelitis complicating Hirschsprung's disease, and also as a sequence to pertussis a fatal pyelitis due to Friedlander's bacillus. He writes that "in future the hematogenous and lymphatic mode of infection will be placed more in the foreground."

Kowitz is practically alone in believing in that the ascending route of infection is uncommon as compared with the hematogenous or lymphatic. He was led to this opinion first by finding that his own cases did not show the customary predominance of girls, in forty cases he had seventeen males to twenty-three females. "This does seem to be such an overwhelming predominance of females that can be spoken of as an essential difference between the sexes." He then discovered in his case the marked relation to the time of the year showing many more cases in late summer and fall.

Clinical Symptoms. Wyman has analyzed carefully the symptoms occurring in his 65 cases and gives the following list:

Vomiting in 39 per cent.
Dysuria in 36 per cent.
Pollakiuria in 40 per cent.
Chills in 15 per cent.
Abdominal in 16 per cent.

Further, he has found that pyelitis occurred as a complication of other diseases as follows:

Otitis media, 8 times; Bronchitis, 2 times; Typhoid, 2 times; Measles, 2 times; Pneumonia, 7 times; Infectious diarrhoea, 4 times; Intestinal Indigestion, 2 times; Tuberculous peritonitis, 1 time.

Gordon separates the cases according to their clinical signs into five groups:

1. Symptoms of general feverish disorders, without any indication that one special symptom was affected.
2. Cerebral symptoms.
3. Pulmonary symptoms.
4. Abdominal symptoms.
5. Urinary symptoms.

Langstein has a similar classification based on the dominating symptom (clinical). That is, a toxic type, a meningeal, a pulmonary, a gastro-intestinal type, one associated with icterus, and one which gives most of the signs of appendicitis. He also mentions a form of pyelitis in which the urine contains many red blood corpuscles and only a few pus cells and says that this "is frequently the cause of erroneous diagnosis."

Thomson and Marsh both emphasize the frequency of chills or rigors, the former says that he does "not remember ever having seen an infant under two years who had a well-marked rigor and who had no pus in his urine"; and the latter that "Shivering is exceedingly rarely met with in infancy, except in this condition."

Stiner mentions redness and swelling of the external genitals, together with inability to urinate, as the initial symptoms of pyelitis which is brought on by exposure to cold.

Urinary Findings. Langstein's hemorrhagic type must be again mentioned, the sediment consisting of "many red corpuscles, only scattered leukocytes, and perhaps here and there a cast. At the time it is difficult to decide whether this is the picture of a hemorrhage nephritis, a calculus or a beginning pyelitis. There are cases which proceed directly to recovery, provided proper medical treatment is instituted at once."

Freeman endeavors to draw a line between a normal and a pathological number of pus cells in urine. According to him, "1-2 leukocytes in a D-field (Zeiss) of precipitate (of centri. fugalized urine) from a female baby does not indicate a pyelitis. That number in a male baby, or more in a female baby, should render the diagnosis suspicious." F. H. Smith has a somewhat similar standard, although he does not find a difference between the sexes. He writes that "it is by no means exceptional to find as few pus cells as 3 to the 1-6 field, and yet the symptoms be most severe."

Kowitz has isolated the infecting organism in forty-four cases and confirms the predominance of the colon group thus:

B. Coli, 24; B. paracoli 10, Staphylococcus (cases of eczema and furunculosis) 3, Mixed 3.

Other authors had nothing new to add to the already well-known urinary picture.

Duration of the Disease. Thompson, Freeman, Marsh, Wilson, and others, agree that the disease may last untreated for several years and show few or no symptoms. It may flare up at any time, however. With correct treatment, Langstein considers that 90 per cent. of patients recover. In severe cases the patient may recover completely after three and one-half years, or the condition may lead to a contracted kidney or to a nephrosis. Marsh gives a favorable prognosis, but adds that "before pronouncing the patient cured, the urine should not only show an absence of organisms and of pus cells, but should also be culturely free."

"In spite of all treatment, many cases become chronic; in these acute exacerbations alternate with long periods of quiescence, but the urine never becomes entirely free from pus cells and organisms."

Treatment. The one form of treatment against which no dissenting voice is raised is the administration of large quantities of fluid. Further than this, there is apparently no specific treatment. In many of the acute cases the patients get well untreated.

Thomson gives sufficient potassium citrate to maintain an alkaline reaction of the urine. Marsh and Wyman agree with this treatment. The latter, however, favors changing the reaction of the urine back and forth by alternate administration of potassium citrate and sodium benzoate.

Freeman says that "the alkaline treatment of pyelitis, while it is safe and will control many cases, is markedly less efficient than other methods of treatment." Langstein goes so far as to say that alkalines are useless in the treatment of the disease. Probably the best of the so-called "urinary antiseptics" is hexamethylenamin. It is pretty generally agreed now that it acts only in an acid medium. Freeman considers it an "absolute antiseptic for certain infections of the urinary tract." He begins always with small doses of from one-half to two grains several times a day, and then increases rapidly, being careful to watch the child and its urine for symptoms of irritation of the kidneys. "Large doses of hexamethylenamin should not usually be given continuously for more than a week at a time, and then several days without any treatment, or with alkaline treatment, it should be started at the maximum dose given before and the amount increased daily until an influence on the urine is obtained. Doses of 25 grains daily in a child of 6 months and 35 to 45 grains a day in a child of 9 to 12 months, may be safely given to some infants."

Wyman as second choice to akalies, gives hexamethylenamine and sodium benzoate. Cannata uses hexamethylenamin alone or alternating with phenyl salicylate (salol), offering at the same time large amounts of an acid drink such as diluted hydrochloric or phosphoric acid. Marsh and Thomson representing the English point of view, are sceptical about the value of hexamethylenamine and phenyl salicylicte. Kowitz considers all urinary antiseptics useless, while Langstein admits that hexamethylenamin may be of value as a prophylactic, but that "in severe cases more is accomplished by salol, in not too small doses (0.6 gm. to 1.0 gm. per diem)."

The value of vaccine therapy is still much disputed. Freeman writing in 1913 of renal infections with colon bacillus: "Vaccines, either autogenous or commercial, are useful in controlling the constitutional symptoms of pyelitis." After another year's experience he adds that they "produce a reduction in the temperature; but so far as I have known, the vaccines do not cure the disease, although they apparently cure it. The child loses its fever and will increase in weight and seem perfectly well, but when the urine is examined one finds the same pus and bacteria as before." Green and Wyman also advice vaccines, but only in resistant cases. Marsh and Kowitz are doubtful about their value. Wilson reports a case in which autogenous vaccines were used without success. The parenchyma of the right kidney finally became involved, abscess formed, and a nephrectomy was done which was followed by prompt recovery. Thomson reports that he had "used vaccines in a few cases, but with disappointing results." Langstein has not only never seen good results from vaccines, but has also "seen a child on the second injection suddenly become very sick, with a high temperature and bloody stools." He considers that this may have been very probably an anaphylactic phenomenon.

REFERENCE.

Thomson, J. London Lancet, 1913, ii, 467.

Wyman, E. T. Boston Med. and Surg. Jour., 1914, clxx., 540.

Smith, F. H. Old Dominion Jour. Med. and Surg., 1914, xix., 77.

Freeman, R. G. Urinary in the Diagnosis and Treatment of Diseases of Childhood, Jour. Am. Med. Assn., Nov. 21, 1914, p. 1802.

Green, R. M. Boston Med. & Surg. Jour., 1913, clviii, 645.

Cannata, S. and Caronia, G. Pediatria, 1914, xxii, No. 9.

Stiner, O. Cor. Bl. f. schweis, Aertz, 1914, xliv Nos. 23-26.

Langstein, L. Med. Klin, 1913, lx., 1491.

Kowitz, H. L. Munchen Med. Wchnschr. June 16, 1914, p. 1341.

Gordon, R. C. Brit. Jour. Child. Disease, 1914, xi, 252.

Marsh, N. P. Brit. Jour. Child Dis., 1913, x, 385.

Wilson, H. W. Brit. Jour. Child Dis., 1913, x, 289

